# P-1218. Title: CAL02, A first in class antivirulence agent, protects Vero cells against Clostridioides difficile toxins

**DOI:** 10.1093/ofid/ofaf695.1411

**Published:** 2026-01-11

**Authors:** Vinolia Chellaraj, Chandana Kruthiventhi, Mike Greenberg, Judith N Steenbergen, Garry Southan, Valentin R Curt

**Affiliations:** Eagle Pharmaceuticals Inc., Woodcliff Lake, NJ; Eagle Pharmaceuticals, Cambridge, Massachusetts; Eagle Pharmaceuticals, Cambridge, Massachusetts; Scientific and Medical Affairs Consulting, LLC, Washington Crossing, Pennsylvania; Eagle Pharmaceuticals Inc, Cambridge, Massachusetts; Eagle Pharmaceuticals Inc., Woodcliff Lake, NJ

## Abstract

**Background:**

*Clostridioides difficile* infection (CDI) is a leading healthcare-associated infection. Clinical and socioeconomic burdens are significant, and CDI is a reportable outcome under the US Centers for Medicare and Medicaid Services Hospital Associated Complications Program. *C. difficile* produces potent toxins, which damage the colon lining, causing inflammation, fluid loss and diarrhea. The need for additional prophylactic and therapeutic options to address CDI is high.

CAL02 is an investigational first-in-class anti-virulence agent composed of proprietary, unilamellar, liposomes, which mimic highly conserved lipid platforms on cell surfaces, acting as a competitive decoy to sequester and inactivate bacterial toxins. CAL02 may improve clinical outcomes of bacterial infections when used as an adjuvant to standard of care treatments.

This study evaluated the ability of CAL02 to protect Vero cells from the toxic effects of *C. difficile* toxins Toxin A and Toxin B.

**Figure 1:**
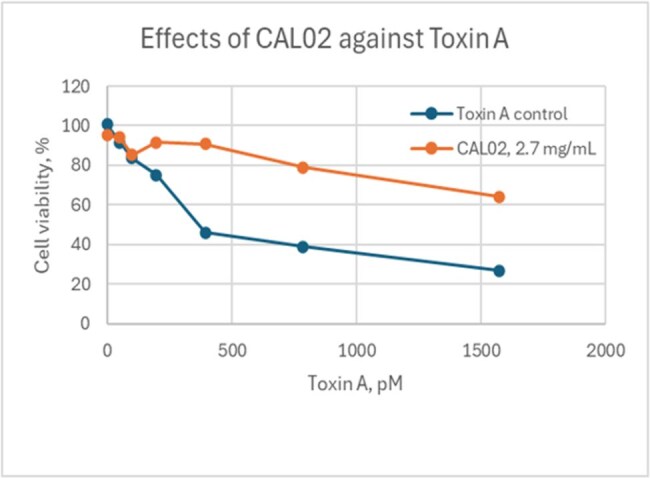
Effects of CAL02 against Toxin A

**Figure 2:**
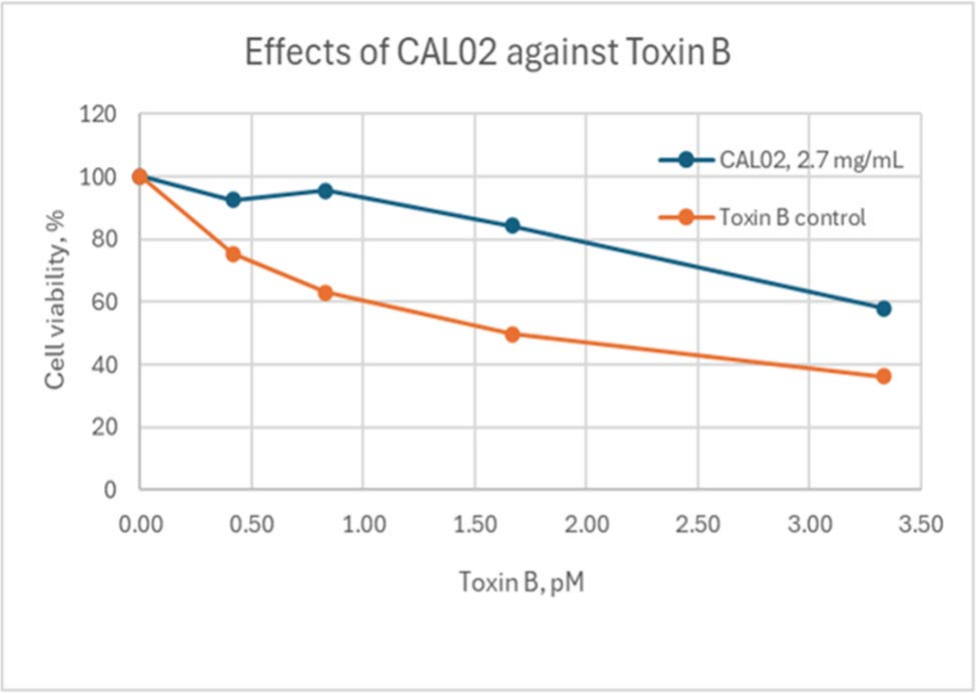
Effects of CAL02 against Toxin B

**Methods:**

The protective effects of CAL02 were studied by treating Vero cells (African Green Monkey Kidney cell line) in 96 well plates with CAL02 at 2.7mg/mL in the presence of increasing concentrations of purified *C.difficile* Toxins A and B . Following an overnight incubation, cell viability was measured using the Alamar blue method.

**Results:**

Toxins used in the study were potent against Vero cells, inducing morphological changes within 3 hours of incubation, with increasing concentrations resulting in increased cell death. In the presence of CAL02 at 2.7mg/ml, the toxic effects of Toxin A and Toxin B on Vero cells were attenuated by up to 83% and 87%, respectively. CAL02 by itself did not have any toxic effects on the viability of the cells.

**Conclusion:**

In the presence of CAL02, the effects of the *C. difficile* toxins on cell morphology and viability were reduced, as observed by light microscopy and Alamar blue assay. Overall, the results are consistent with CAL02 acting as a decoy mechanism and sequestering the toxins, thereby preventing them from destroying the cells. These results warrant further investigation into the potential role of CAL02, a novel antivirulence agent, in the treatment of CDI symptoms.

**Disclosures:**

Vinolia Chellaraj, M. Sc, Eagle Pharmaceuticals Inc: Employee|Eagle Pharmaceuticals Inc: Stocks/Bonds (Public Company) Chandana Kruthiventhi, M.S., Eagle Pharmaceuticals: Employee Mike Greenberg, MD, Eagle Pharmaceuticals: Employee|Eagle Pharmaceuticals: Stocks/Bonds (Public Company) Judith N. Steenbergen, PhD, AcurX: Advisor/Consultant|Basilea: Advisor/Consultant|Bioversys: Advisor/Consultant|Clarametyx: Advisor/Consultant|Eagle Pharmaceuticals: Advisor/Consultant|F2G: Advisor/Consultant|Genentech: Advisor/Consultant|Innoviva: Advisor/Consultant|Meitheal: Advisor/Consultant|Melinta: Advisor/Consultant|Neuraptive: Advisor/Consultant|Neuraptive: Advisor/Consultant|Roche: Advisor/Consultant|Wockhardt: Advisor/Consultant Garry Southan, PhD, Eagle Pharmaceuticals, inc: Employee Valentin R. Curt, MD, Eagle Pharmaceuticals: I am an employee of Eagle Pharmaceuticals|Eagle Pharmaceuticals: Ownership Interest|Eagle Pharmaceuticals: Stocks/Bonds (Public Company)

